# Transcriptome analysis and identification of genes associated with fruiting branch internode elongation in upland cotton

**DOI:** 10.1186/s12870-019-2011-8

**Published:** 2019-10-07

**Authors:** Feiyan Ju, Shaodong Liu, Siping Zhang, Huijuan Ma, Jing Chen, Changwei Ge, Qian Shen, Xiaomeng Zhang, Xinhua Zhao, Yongjiang Zhang, Chaoyou Pang

**Affiliations:** 1 State Key Laboratory of Cotton Biology (Hebei Base)/College of Agronomy, Hebei Agricultural University, Baoding, 071001 Hebei China; 2 State Key Laboratory of Cotton Biology, Institute of Cotton Research, Chinese Academy of Agricultural Sciences, Anyang, 455112 Henan China

**Keywords:** Cotton, Plant architecture, RNA-Seq, Internode elongation, Plant hormone, TFs

## Abstract

**Background:**

Appropriate plant architecture can improve the amount of cotton boll opening and allow increased planting density, thus increasing the level of cotton mechanical harvesting and cotton yields. The internodes of cotton fruiting branches are an important part of cotton plant architecture. Thus, studying the molecular mechanism of internode elongation in cotton fruiting branches is highly important.

**Results:**

In this study, we selected internodes of cotton fruiting branches at three different stages from two cultivars whose internode lengths differed significantly. A total of 76,331 genes were detected by transcriptome sequencing. By KEGG pathway analysis, we found that DEGs were significantly enriched in the plant hormone signal transduction pathway. The transcriptional data and qRT-PCR results showed that members of the *GH3* gene family, which are involved in auxin signal transduction, and *CKX* enzymes, which can reduce the level of CKs, were highly expressed in the cultivar XLZ77, which has relatively short internodes. Genes related to ethylene synthase (*ACS*), *EIN2/3* and *ERF* in the ethylene signal transduction pathway and genes related to *JAR1*, *COI1* and *MYC2* in the JA signal transduction pathway were also highly expressed in XLZ77. Plant hormone determination results showed that the IAA and CK contents significantly decreased in cultivar XLZ77 compared with those in cultivar L28, while the ACC (the precursor of ethylene) and JA contents significantly increased. GO enrichment analysis revealed that the GO categories associated with promoting cell elongation, such as cell division, the cell cycle process and cell wall organization, were significantly enriched, and related genes were highly expressed in L28. However, genes related to the sphingolipid metabolic process and lignin biosynthetic process, whose expression can affect cell elongation, were highly expressed in XLZ77. In addition, 2067 TFs were differentially expressed. The *WRKY*, *ERF* and *bHLH* TF families were the top three largest families whose members were active in the two varieties, and the expression levels of most of the genes encoding these TFs were upregulated in XLZ77.

**Conclusions:**

Auxin and CK are positive regulators of internode elongation in cotton branches. In contrast, ethylene and JA may act as negative regulators of internode elongation in cotton branches. Furthermore, the *WRKY*, *ERF* and *bHLH* TFs were identified as important inhibitors of internode elongation in cotton. In XLZ77(a short-internode variety), the mass synthesis of ethylene and amino acid conjugation of auxin led to the inhibition of plant cell elongation, while an increase in JA content and degradation of CKs led to a slow rate of cell division, which eventually resulted in a phenotype that presented relatively short internodes on the fruiting branches. The results of this study not only provide gene resources for the genetic improvement of cotton plant architecture but also lay a foundation for improved understanding of the molecular mechanism of the internode elongation of cotton branches.

**Electronic supplementary material:**

The online version of this article (10.1186/s12870-019-2011-8) contains supplementary material, which is available to authorized users.

## Background

Cotton (*Gossypium hirsutum* L.) is grown worldwide and is an important fiber crop [1]. Appropriately increasing the planting density of cotton plants represents an effective method for increasing cotton yields. However, broad and loose cotton plant architecture has become the key factor limiting increased cotton planting density. Therefore, appropriate plant architecture and colony structure are important for the cultivation of high-yielding cotton.

Plant architecture is a comprehensive representation of plant morphology and structure, physiological and ecological functions, etc. Plant architecture is a key determinant of light reception, photosynthate production, and nutrient partitioning and plays an important role in crop yield, product quality, and cultivation management [2]. In higher plants, formation of plant architecture also encompasses plant morphology-related organs throughout the whole growth and development of the plants, especially the formation, shape and location of the branches, leaves and flowers [3]. Overall, cotton plant architecture comprises several growth components, such as the height of the main stem, both the number and length of the fruiting branches and roots, the internode length of both the main stem and fruiting branches, and the distribution of cotton bolls throughout the whole plant, as well as the cotton growth structure and boll formation. The effects of plant architecture on lint yield are especially important [4]. Architecture can significantly affect the light distribution within and penetration into a crop canopy and thus can alter plant growth, biomass partitioning, boll distribution, and yield potential [5].

Plant hormones such as auxin, gibberellins (GAs), brassinolide and cytokinins (CKs) play important roles in the process of plant type formation; specifically, these hormones play important roles in the process of plant dwarfing, stem-leaf angle determination, and lobulation, while ethylene (ET) and abscisic acid (ABA) exert many inhibitory effects [6, 7]. Plant height is determined by several developmental factors; specifically, stem elongation due to cell division and expansion (EXP) of both the shoot apical meristem (SAM) and the intermediate meristem play decisive roles. Stem elongation is controlled by several hormones, including GAs, brassinosteroids, auxin, and strigolactones (SLs) [8]. Relatively little is known about the molecular mechanisms controlling the growth of intercalary meristems, but this process is triggered by ET and promoted by GA [9]. In the case of deepwater rice, GA induces internode growth by promoting both cell division and cell elongation [10]. *Oscen1* and *Oscen2*, which are members of the *TERMINAL FLOWER 1* (*TFL1*)*/CENTRORADIALIS* (*CEN*) gene family in rice, exhibit distinct expression patterns mainly in secondary meristems. Overexpression of *Oscen1* and *Oscen2* in transgenic rice plants results in increased numbers of shorter internodes, suggesting that these genes regulate the development of basic structures by stimulating the activities of secondary meristems in the uppermost phytomers [11]. The downregulation of GA biosynthesis-related genes in cotton inhibits cell elongation, reduces plant height and shortens internode lengths, which indicates that GA has an important effect on internode elongation of the main stem in cotton plants [1].

In terms of shoot architecture, the SAM determines plant phyllotaxy and impacts axillary meristem (AM) formation. During leaf development, an AM can develop in the axil and subsequently give rise to a secondary shoot. Lateral shoot outgrowth is fundamentally important for controlling shoot architecture [8]. By locating and cloning the *GbAF* gene (which controls the axillary flowering phenotype) of *Gossypium barbadense* L. and the *GhCB* gene (which controls the clustered boll phenotype) of *Gossypium hirsutum* L., researchers observed that different phenotypes were caused by mutations at different SNP loci at the same locus, which is homologous with *SELF-PRUNING* (*SP*) in tomato; moreover, silencing *GoSP* in *Gossypium barbadense* L. and *Gossypium hirsutum* L. transformed the plant architecture such that plant growth was limited [12]. The above mentioned studies focused mainly on the elongation of the internode of both the main stem and zero-type fruiting branches. No studies have investigated the physiological and transcriptional regulatory mechanisms that govern the internode elongation of indefinite fruiting branches of cotton.

In this study, two cotton plant varieties whose fruiting branch lengths significantly differ but whose heights do not significantly differ were used to identify and analyze the genes and related metabolic pathways involved in internode elongation via RNA Sequencing (RNA-Seq) techniques. The endogenous hormone contents and analysis of both transcription factors (TFs) and related genes revealed the molecular mechanism of internode elongation of cotton fruiting branches. Together, the results provide a valuable resource for further identification of genes related to internode elongation of cotton branches.

## Results

### Differences in the internode lengths of fruiting branches between genotypes

**Fig. 1:**
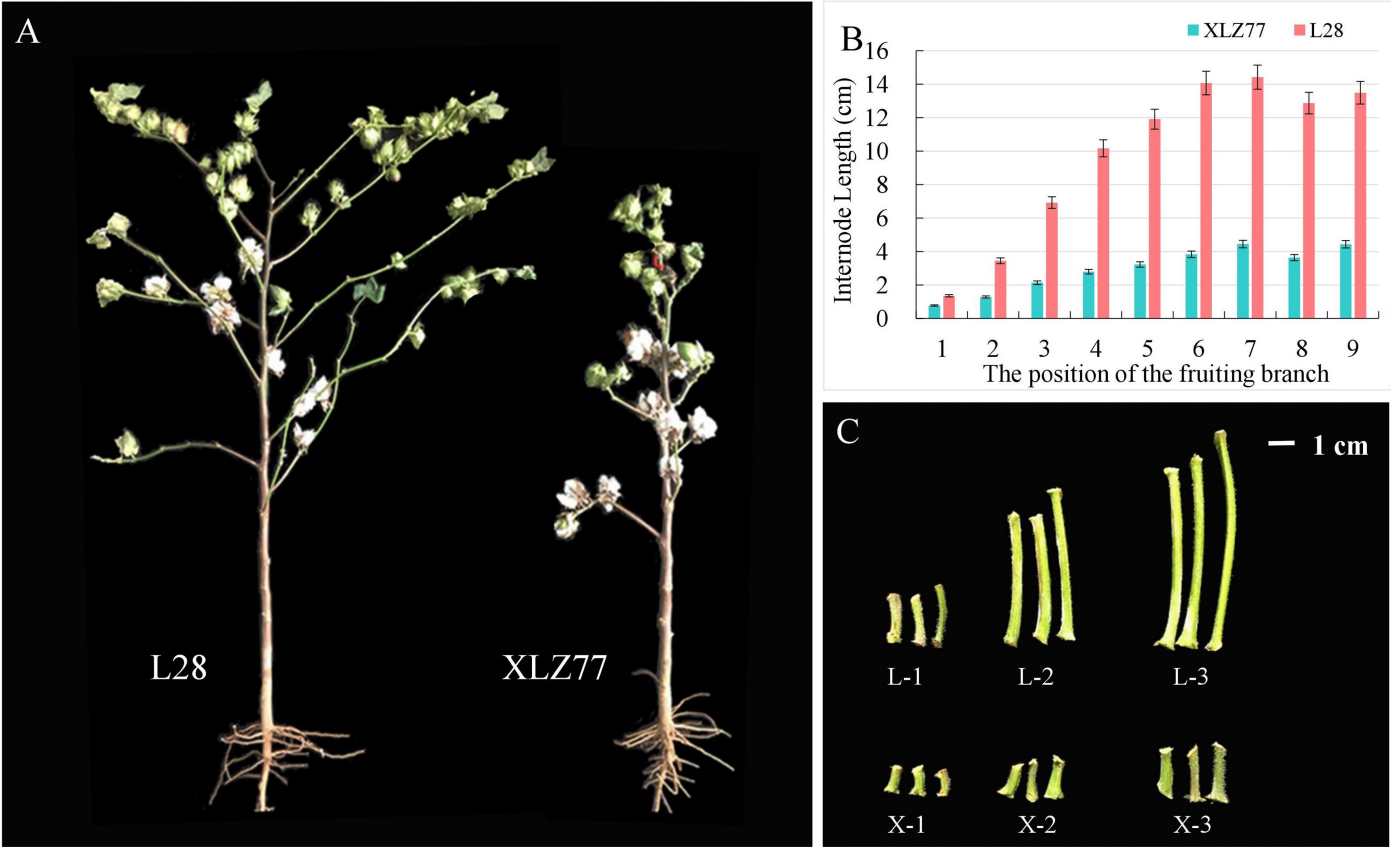
Phenotypic difference between XLZ77 and L28. **a**. Whole-plant phenotypic differences between XLZ77 and L28. **b**. The first internode length of the fruiting branch of the two genotypes; the position of the fruiting branch is counted from the top of the plant. **c**. Sample differences between XLZ77 and L28. L, L28. X, XLZ77. The numbers represent the position of the fruiting branch

The differences in plant architecture were very significant between the two varieties (Fig. 1a). The L28 variety had longer fruiting branch internodes and loose plant architecture; the XLZ77 variety had shorter fruiting branch internodes and compact plant architecture. However, there was no significant difference in plant height between the two varieties. We measured the length of the first internode of each branch of both varieties (Fig. 1b); the internode of L28 gradually elongated until reaching the sixth branch, after which the length essentially stabilized at approximately 14 cm. On the other hand, the internode length of XLZ77 was stabilized at approximately 3 cm at the fourth branch. To explain the differences in internode lengths of the fruiting branches, we selected the first internode of the first, second and third branches from the top for transcriptome sequencing (Fig. 1c).

### RNA-Seq analysis and gene annotation

Approximately 1.05 × 10^9^ raw reads of 150 bp paired-end reads were generated from the eighteen samples via RNA-Seq. After 0.42% adaptor deletion, 1.30% low-quality read filtering and 0.001% N-containing read filtering, > 98.27% of the sequences were confirmed as clean reads. Detailed information about the obtained reads is listed in Additional file 1. By anchoring the clean reads to the cotton reference genome, we obtained the expression data of 76,331 genes among the 18 samples (Additional file 2). The BLAST algorithm was then used to identify *Arabidopsis thaliana* transcripts to determine the functional annotations in cotton.

Screening and identification of differentially expressed genes (DEGs).

To identify DEGs between the two genotypes, the edgeR package (http://www.rproject.org/) was used. We identified genes fold change (FC) was ≥2 and whose false discovery rate (FDR) was < 0.05 in a comparison as significantly differentially expressed. The number of DEGs between each pair of compared groups is shown in Additional file 3. When the different internodes of the same variety were compared, the numbers of upregulated and downregulated DEGs in L-1 vs L-2 and in L-1 vs L-3 were nearly identical. The fewest DEGs occurred in the X-1 vs X-2 comparison, but in the X-1 vs X-3 comparison, the number of upregulated DEGs in X-1 was 1.8 times as high as that of downregulated DEGs. However, when the same internodes of the different varieties were compared, the number of upregulated DEGs in X-1 was 3.5 times as high as that of the downregulated DEGs in the X-1 vs L-1 comparison. In the X-2 vs L-2 comparison, the number of upregulated DEGs in X-1 was 3.1 times as high as that of the downregulated DEGs.

### Kyoto encyclopedia of genes and genomes (KEGG) enrichment analysis showed that plant hormones played important roles in internode elongation of fruiting branches in cotton

To understand the DEGs functions, KEGG pathway enrichment analysis was performed in accordance with a *p*-value of 0.05 adjusted by the FDR as the cutoff. The analysis results are shown in Additional file 4. Plant hormone signal transduction pathways were significantly enriched in the X-1 vs L-1, X-3 vs L-3, L-1 vs L-2 and L-1 vs L-3 comparisons, which suggests that plant hormone signal transduction pathways may play important roles in the internode elongation of fruiting branches in cotton.

To identify the contributions of hormone-mediated transcriptional regulation to the internode elongation of fruiting branches in cotton, we mapped the DEG transcripts to eight hormone-related pathways in the Arabidopsis Hormone Database; 1009 genes associated with various aspects of hormone homeostasis were enriched (Additional file 5). The genes whose expression significantly differed the most were related to auxin, ET, CK and jasmonic acid (JA).

**Fig. 2:**
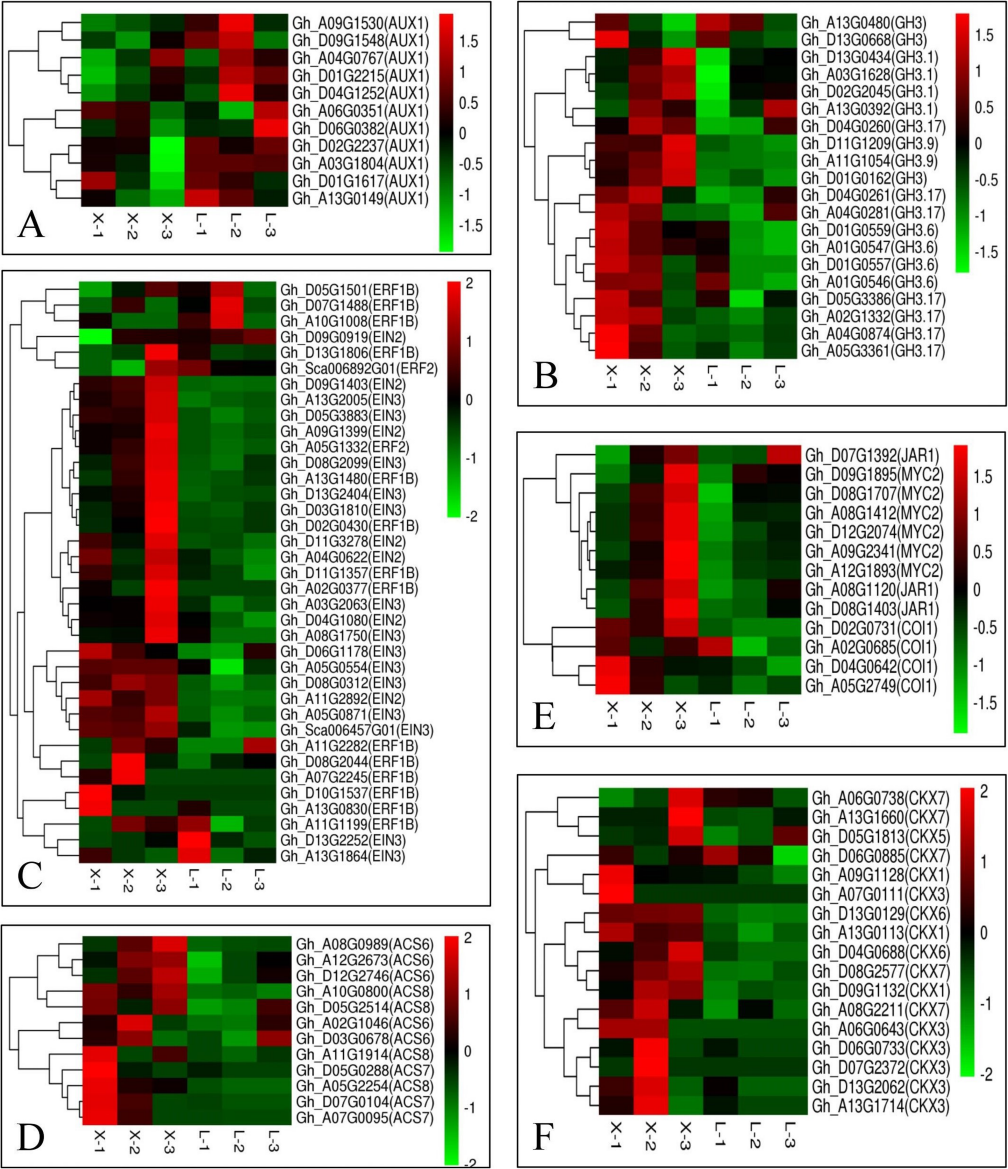
Cluster heat map of plant hormone-related genes. **a**. AUX1; **b**. GH3; **c**. EIN2/3 and ERF1/2; **d**. ACS; **e**. JAR1, MYC2, COI1; **f**. CKX. The expression of all the genes listed in these maps is shown in Additional file 6

Auxin influx carrier (*AUX1*) is a high-affinity indole-3-acetic acid (IAA) importer. In the present study, a total of 18 *AUX1*-related genes were differentially expressed in the two varieties. Eleven of these genes were highly expressed in L28 (Fig. 2a). *GH3* is a negative regulator of auxin signal transduction. A total of 49 DEGs between both varieties were annotated as *GH3*, 20 of which were highly expressed in XLZ77 (Fig. 2b); we excluded 29 genes with FPKM < 1.

In the ET signal transduction pathway, a total of 7 DEGs between the varieties were annotated as ethylene-insensitive protein (*EIN2*), 20 DEGs were annotated as *EIN3* and 35 DEGs were annotated as ethylene response factor (*ERF*)*1/2*. All of the *EIN2*-related and 14 of the *EIN3*-related DEGs were highly expressed in XLZ77, while most of the *ERF1/2*-related genes were also highly expressed in XLZ77 (Fig. 2c). In addition, ACC synthase (*ACS*) is the most important enzyme in the ET synthesis pathway. There were 26 DEGs related to *ACS* between the two varieties, excluding unexpressed genes, 12 of which were highly expressed in XLZ77 (Fig. 2d). All of the above results indicate that the genes involved in ET synthesis and signal transduction were highly expressed in XLZ77.

*Jasmonate synthetase* (*JAR1*)*, insensitive mutant of coronatine, jasmonate SCF-COI1 receptor complex* (*COI1*) and *MYC2* are all positive regulators of the JA signal transduction pathway. In our results, a total of 8 DEGs between the varieties were annotated as *JAR1*, 10 DEGs were annotated as *COI1* and 13 DEGs were annotated as *MYC2*. The majority of these genes were highly expressed in XLZ77 (Fig. 2e), and we excluded the genes with FPKM < 1 and newly annotated genes.

Degradation of the GK plant hormones is catalyzed by the cytokinin oxidase/dehydrogenase (*CKX*) enzymes. In the present study, 27 DEGs related to *CKX* were significantly enriched between the two varieties, the majority of which were highly expressed in XLZ77 (Fig. 2f).

Endogenous hormone contents in the two genotypes.

**Fig. 3:**
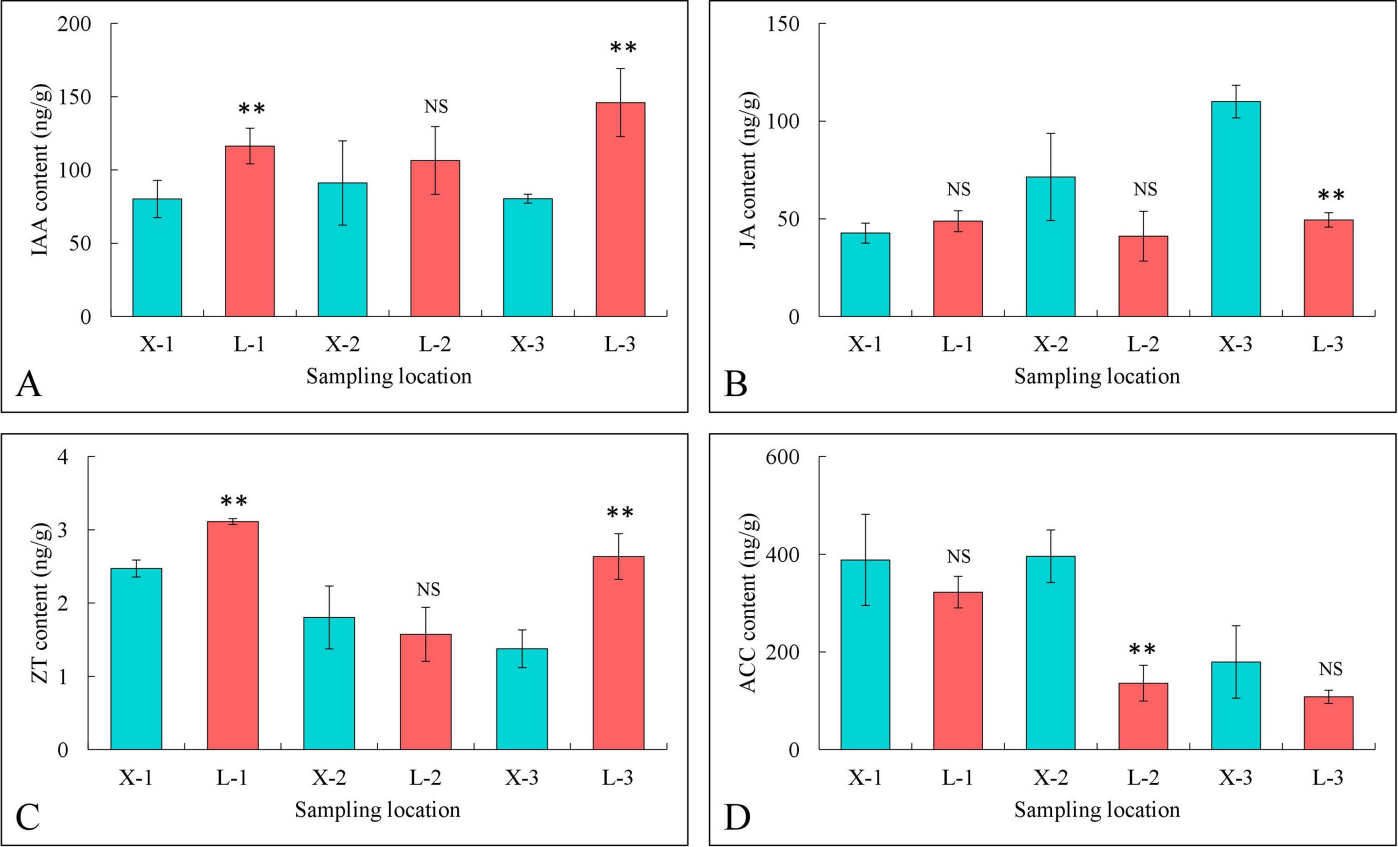
Endogenous hormone contents in XLZ77 and L28. **a**. IAA; **b**. JA; **c**. ZT; **d**. ACC. The data were analyzed by three independent repeats, and standard deviations are shown with error bars. The internodes in the same position act as a comparison group. Significant differences are indicated by “*”, “NS” represents no significant difference

To compare the roles of endogenous hormones in internode elongation between different internodes of cotton fruiting branches, the contents of IAA, zeatin (ZT) and JA were measured. Independent samples collected from the first internodes of the first, second and third branches from the top were used for endogenous hormone measurements. As shown in Fig. 3, the content of IAA between the two varieties significantly differed at the inverted 1 and 3 internodes. The content of JA in the inverted 3 internode of XLZ77 was significantly higher than that in L28 and reached 107.75 ng/g. Furthermore, compared with that in XLZ77, ZT content in L28 increased by 0.64 ng/g and 1.26 ng/g in inverted 1 and 3 internodes, respectively; these differences were significant. In addition, we measured the content of the ET precursor ACC, which was higher in all three internodes of XLZ77 relative to L28, and the difference was significant at the inverted 2 internode.

### Validation of genes related to plant hormone signal transduction by qRT-PCR

**Fig. 4:**
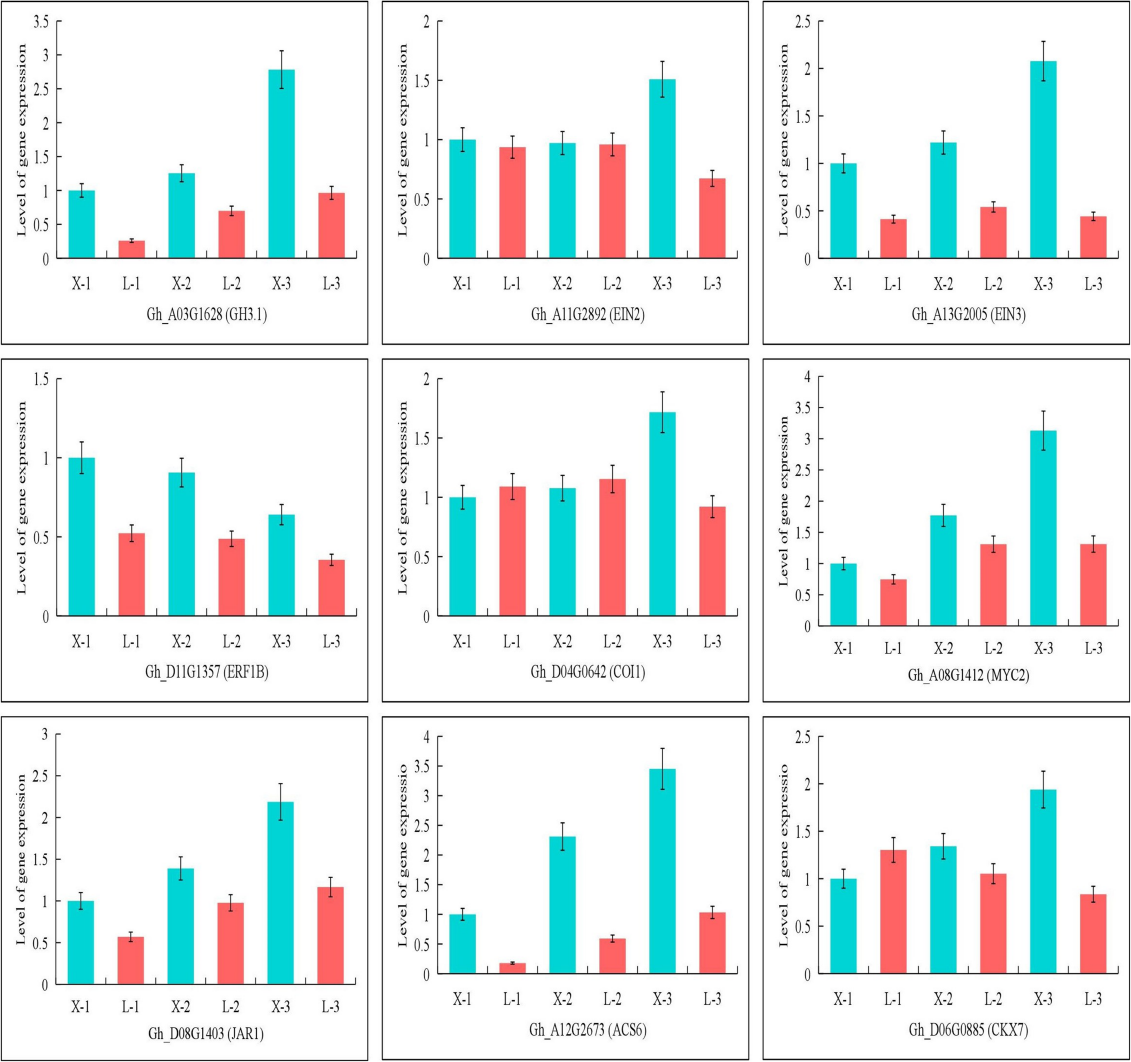
RT-PCR validation of genes related to plant hormone signal transduction. Expression levels of 12 plant hormone signal transduction-related genes in the two varieties were validated by qRT-PCR. All data are based on the analysis of three independent biological repeats

To further verify the correctness of the RNA-Seq analysis results, twelve genes whose expression was relatively high were selected for real-time quantitative PCR (qRT-PCR). The qRT-PCR results for these genes were highly consistent with the RNA-Seq data (Fig. 4). With the exception of AUX1, all other genes were highly expressed in the short-internode variety XLZ77. Here, we show only nine pictures, and the other three pictures are shown in Additional file 7. The validation experiments support the accuracy of the relative values provided by the RNA-Seq analysis.

### Gene ontology (GO) enrichment analyses of DEGs

GO category enrichment analysis was performed using the DEGs identified from each comparison. Here, our analysis includes only the majority of the GO terms associated with biological processes (Additional file 8).

With respect to the X-1 vs L-1 comparison group, DEGs were significantly enriched in the GO categories of nucleosome assembly, chromatin assembly and nucleosome organization. With respect to the X-2 vs L-2 comparison group, DEGs were significantly enriched in the GO categories of nucleosome assembly, phospholipid metabolic process and membrane lipid metabolic process. In the X-3 vs L-3 comparison group, DEGs were significantly enriched in the GO categories of mitotic cell cycle process, cell cycle, cell division, the secondary metabolic process and cell wall organization. With respect to the L-1 vs L-2 comparison group, the DEGs were significantly enriched in the xyloglucan metabolic process, cell wall organization, cell wall organization or biogenesis and cell wall polysaccharide metabolic process GO categories. With respect to the L-1 vs L-3 comparison group, DEGs were also significantly enriched in the GO categories of cell wall organization or biogenesis, xylan biosynthetic process, xylan metabolic process and plant-type cell wall loosening. With respect to the X-1 vs X-2 comparison group, DEGs were significantly enriched in the GO categories of plant-type cell wall organization or biogenesis, xyloglucan metabolic process, cell wall modification and plant-type cell wall loosening. In addition, the majority of genes related to these GO categories were highly expressed in X-2. However, with respect to the X-1 vs X-3 comparison group, DEGs were significantly enriched in the GO categories of cell cycle, regulation of cell cycle, cell division, cell wall biogenesis and secondary metabolic process.

### Cluster analysis of DEGs and GO category enrichment analysis of each module

**Fig. 5:**
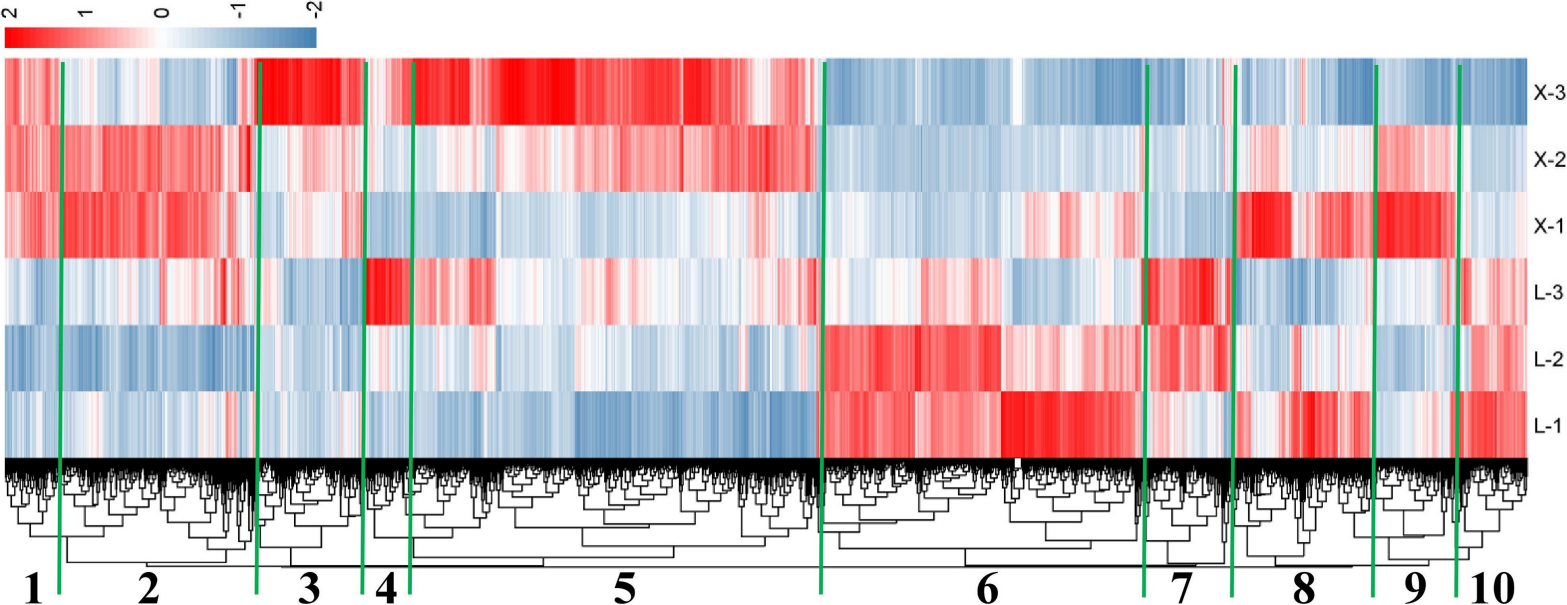
Cluster analysis of 7330 DEGs. According to the gene expression trends, the cluster heat map was divided into 10 modules to further explore the biological significance of each module. The number in the figure represents the name of each module

In accordance with the condition of FPKM ≥10 for at least one sample, we screened 7330 DEGs in the comparison groups X-1 vs L-1, X-2 vs L-2, X-3 vs L-3, etc., for cluster analysis (Fig. 5). Based on the differences in the expression trends shown in the cluster heat map, the 7330 DEGs were divided into 10 modules, each with a unique expression trend. GO category enrichment analysis was performed on the DEGs in each module. According to the enrichment analysis and gene annotation results, we identified important GO terms for each module. The number of genes in each module and information for selected important GO terms are shown in Table 1, and the expression of the genes in each module is shown in Additional file 9. Here, we analyzed only the majority of GO terms based on biological processes (Additional file 10).

**Table 1:** Number of genes in each module and related GO term information

Module Name	Number of Genes	Related GO ID	Description	Number of GO Genes
Module1	263	GO:0006629	lipid metabolic process	23
Module2	987	GO:0043624	cellular protein complex disassembly	14
		GO:0006665	sphingolipid metabolic process	8
Module3	502	GO:0009809	lignin biosynthetic process	14
Module4	175	GO:0046271	phenylpropanoid catabolic process	13
Module5	2003	GO:0009873	ethylene-activated signaling pathway	76
Module6	1533	GO:0006334	nucleosome assembly	28
Module7	454	GO:0071555	cell wall organization	51
Module8	633	–	–	–
Module9	440	–	–	–
Module10	340	GO:0010410	hemicellulose metabolic process	10

With respect to module 1, the expression of genes was higher in XLZ77 than in L28. The DEGs were significantly enriched in the lipid metabolic process GO category. The genes *Gh_A03G0871* and *Gh_D02G1254* were mapped to *AT1G14290*, which encodes sphingoid base hydroxylase 2 (*SBH2*) and is involved in sphingolipid trihydroxy long-chain base (4-hydroxysphinganine) biosynthesis.

With respect to module 2, the gene expression in X-2 and X-3 was higher in XLZ77 than in L28. DEGs were significantly enriched in the GO categories of cellular protein complex disassembly, protein complex disassembly, cell wall biogenesis and sphingolipid metabolic process. The gene *Gh_D09G0602* was significantly enriched in the above GO categories, was expressed more than the other genes, it was mapped to *AT3G16630*, which encodes *KINESIN-13A*. There are two important genes in the metabolic process of sphingolipids category: *Gh_D02G1253* and *Gh_A11G3137*. The gene *Gh_D02G1253* was mapped to *AT1G14290*, which encodes the sphingosine hydroxylase *SBH2*. The gene *Gh_A11G3137* was mapped to *AT5G10480*, which encodes the protein tyrosine phosphatase *PAS2*.

With respect to module 3, gene expression was highest in X-3. The first GO category enriched was the lignin biosynthetic pathway, which included 14 genes: *Gh_D11G2151* and *Gh_D12G0788* were mapped to *AT2G22420*, which encodes a peroxidase superfamily protein (*POX*); *Gh_D10G2466* and *XLOC_076267* were mapped to *AT2G38080*, which encodes a laccase/diphenol oxidase family protein (*LAC4*); *Gh_A10G1518* and *Gh_D10G1769* were mapped to chitinase-like protein 2 (*CTL2*); *Gh_A04G1032* was mapped to *AT4G34050*, which encodes an S-adenosyl-L-methionine-dependent methyltransferase superfamily protein (*CCOAOMT1*); *Gh_A05G1579* was mapped to *AT4G39330*, which encodes cinnamyl alcohol dehydrogenase 9 (*CAD*); and *Gh_A11G1648*, *Gh_D11G1805* and *Gh_Sca004990G01* were mapped to *AT4G36220*, which encodes ferulic acid 5-hydroxylase 1 (*F5H*). All of the above proteins (enzymes) are important in the biosynthesis of lignin.

Regarding module 4, the expression of genes was highest in L-3. The first GO category enriched was phenylpropanoid catabolic process, which contrasts with the GO category results obtained for module 3. A total of 13 genes were enriched in this module, and all of these genes were related to *LAC*: *LAC2*, *LAC4*, *LAC5* and *LAC17*. The results showed that lignin was degraded more in L28 than in XLZ77; but decomposed and was metabolized to a lesser degree in the latter. This finding explains the differences in internode length.

With respect to module 5, the majority of genes were highly expressed in X-3. The DEGs were significantly enriched in the ET-activated signaling pathway GO category. A total of 76 genes were enriched in this module; most of these genes were related to *ERFs*. In addition, 8 genes in the pathway were mapped to *AT3G51550*, which encodes a *FERONIA* protein, a member of a family of receptor-like protein kinases.

Regarding module 6, gene expression in the three internodes was higher in L28 than in XLZ77. Moreover, the expression of these genes decreased as the internodes of fruiting branches elongated for the same variety. The DEGs were significantly enriched in the nucleosome assembly GO category. A total of 28 genes were enriched in this module; 19 of these genes were mapped to *AT5G65360*, which encodes a histone superfamily protein. In addition, most of the GO categories in module 6 were related to nucleosome assembly, chromatin assembly, DNA replication, mitosis, etc. It can be inferred that the genes were mainly enriched in the pathway of cell division.

With respect to module 7, the gene expression of the three internodes was higher in L28 than in XLZ77. In addition, the expression of these genes increased in L28 as the internodes of the fruiting branches elongated. The first GO category enriched was cell wall organization, which included 51 genes, 9 of which were mapped to *EXP*-related genes encoding expansins. In our samples, 107 *EXP*-related genes were screened from among all DEGs, and the expression of the majority of these genes was higher in L28 than in XLZ77. In addition, three genes were mapped to the *AT1G75500* gene, which encodes the *WALLS ARE THIN 1* (*WAT1*) gene.

With respect to module 10, gene expression in the three internodes was higher in L28 than in XLZ77. The expression of most of these genes was relatively high in L-1. DEGs were significantly enriched in the hemicellulose metabolic process GO category. A total of 10 genes were enriched in this category; 8 of the genes were mapped to xyloglucan endotransglucosylase/hydrolase (*XTH*)-related genes. Three genes mapped to *AT4G03210*, which encodes *XTH9*. It can be inferred that the high expression of *XTH* genes in L28 leads to cell expansion and elongation.

Important genes and their predicted functions in the internode elongation of fruiting branches in cotton are shown in Table 2. These genes could be used to genetically improve internode length in cotton.

**Table 2:** Important genes and their predicted functions in the internode elongation of fruiting branches in cotton

Gene ID	L-1_fpkm	L-2_fpkm	L-3_fpkm	X-1_fpkm	X-2_fpkm	X-3_fpkm	Description	Predicted Function	Reference
Gh_A03G0871	140.59	123.31	174.71	288.19	307.84	312.05	Sphingoid base hydroxylase 2	Inhibition of growth, Promote apoptosis	Chen M et al., 2008 [13]
Gh_D02G1254	51.05	42.47	73.17	193.67	193.92	168.60	Sphingoid base hydroxylase 2	Inhibition of growth, Promote apoptosis	Chen M et al., 2008 [13]
Gh_D02G1253	26.06	4.56	3.80	64.51	24.95	22.93	Sphingoid base hydroxylase 2	Inhibition of growth, Promote apoptosis	Chen M et al., 2008 [13]
Gh_D09G0602	56.28	47.55	51.27	109.19	97.56	71.71	Kinase-13A	Inhibition of cell expansion	Fujikura U et al., 2014 [14]
Gh_D09G0602	56.28	47.55	51.27	109.19	97.56	71.71	Kinase-13A	Inhibition of cell cell size	Li YJ et al., 2017 [15]
Gh_A11G3137	4.98	2.37	7.81	10.01	10.12	3.79	Protein-tyrosine phosphatase-like, PTPLA	Inhibition of cell division	Bach L et al., 2008 [16]
Gh_A05G1579	23.01	28.32	29.73	24.85	28.62	57.19	Cinnamyl alcohol dehydrogenase 9	Lignin accumulation	Mansell RL et al., 2014 [17]
Gh_A10G1518	14.25	16.39	79.99	12.18	20.41	209.74	Chitinase-like protein 2	Accelerate secondary metabolism	Hossain MA et al., 2010 [18]
Gh_D10G1769	11.60	13.33	67.11	7.79	14.97	144.79	Chitinase-like protein 2	Accelerate secondary metabolism	Hossain MA et al., 2010 [18]
Gh_A11G2936	14.46	20.28	55.22	7.95	9.54	40.63	Laccase/diphenol oxidase family protein	Promotion of lignin decomposition	Berthet S et al., 2011 [19]
Gh_D03G1128	19.76	29.29	52.91	20.38	28.83	47.27	Laccase/diphenol oxidase family protein	Promotion of lignin decomposition	Zhao Q et al., 2013 [20]
Gh_D09G2057	21.45	62.35	73.01	33.54	70.74	107.94	Malectin/receptor-like protein Kinase family protein	Protoplast alkalization	Barbez E et al., 2017 [21]
Gh_D10G0981	237.30	215.92	111.36	116.78	90.66	36.42	Histone superfamily protein	Promoting cell division	Günesdogan U et al., 2014 [22]
Gh_A08G2114	202.08	170.28	81.50	94.56	62.85	30.06	Histone superfamily protein	Promoting cell proliferation	Günesdogan U et al., 2014 [22]
Gh_A13G0050	127.76	214.03	257.34	121.15	156.57	118.64	Expansin A8	Promote cell loosening and expansion	Nardi CF et al., 2014 [23]
Gh_D13G0060	196.61	288.97	352.52	199.21	232.59	172.40	Expansin A8	Promote cell loosening and expansion	Nardi CF et al., 2014 [23]
Gh_D01G0964	47.14	51.81	75.14	36.00	37.19	32.03	Walls Are Thin 1	Promoting cell elongation	Ranocha P et al., 2010 [24]
Gh_A01G0922	36.58	39.66	58.01	24.81	25.32	28.10	Walls Are Thin 1	Promoting cell elongation	Ranocha P et al., 2010 [24]
Gh_A03G1432	102.22	86.74	92.84	53.86	58.35	44.55	Xyloglucan endotransglucosylase/hydrolase 9	Promoting cell proliferation and elongation	Shin YK et al., 2006 [25]
Gh_A13G0500	39.81	26.30	26.26	13.99	14.66	12.00	Xyloglucan endotransglucosylase/hydrolase 9	Promoting cell proliferation and elongation	Shin YK et al., 2006 [25]

### Cluster analyses of differentially expressed TFs

TFs are key regulatory proteins that are essential for the regulation of gene expression. There were significant differences in the expression of 2067 TFs between the two varieties (Additional file 11). The *WRKY*, *ERF* and *bHLH* families were the top three largest families of TFs. As shown in Table 3, 224 genes in the *WRKY* family, 256 genes in the *bHLH* family and 399 genes in the *ERF* family were enriched between the two varieties; most of them were upregulated in XLZ77. The high expression of these TF families in XLZ77 could be the main reason for the shorter internodes of the fruiting branches.

**Table 3:** Number of TFs

TF	Number	Upregulated	Downregulated
bHLH	256	158	98
WRKY	224	176	48
ERF	399	230	169

## Discussion

### Regulation of plant hormone involved in the internode elongation of fruiting branches in cotton

Higher plants develop their plant architecture by regulating the activity of the apical meristem and side meristem. The activity of the meristems is regulated by environmental signals, developmental stages and genetic factors. The comprehensive regulation of these factors confers developmental plasticity to plants and their adaptability to the environment. Plant hormones are at the center of a network system that governs many regulatory signals. These hormones play an important role in plant architecture formation [26]. Specifically, the phytohormones auxin and CK can promote cell expansion and division, whereas ET and JA inhibit organ growth by affecting cell expansion [27].

The plant hormone IAA controls growth and developmental responses throughout the life of a plant; specifically, IAA is involved in cell expansion and division, tissue differentiation, organ development and a variety of physiological responses [28]. The content and distribution of auxin play important roles in plant morphogenesis [29]. Auxin is a weak acid that protonates in a low-pH environment outside the cell and enters the cell by infiltration or mediation by an input vector. After auxin enters a cell, the increased pH of the cytoplasm causes the auxin to remain in the cell in an ionic state, whereas auxin is transported out of the cell depending on specific output carrier. The *AUX1* gene encodes a component of the auxin influx carrier, and mutations within *AUX1* selectively impair the action of auxins that require carrier-mediated uptake [30]. The accumulation of auxin in the root apex clearly decreases when *AUX1* is mutated [31]. The *Gretchen Hagen-3* (*GH3*) gene family encodes a class of luciferase [32] that can catalyze the formation of an inactive form of auxin by binding IAA and amino acid molecules in vitro [33]; this luciferase is a negative regulator of auxin signal transduction. Overexpression of *GH3.2*, *GH3.5* or *GH3.6* can result in plant dwarfism, inhibition of hypocotyl elongation of seedlings, reduction in lateral roots, loss of apical dominance and endogenous auxin deficiency [34]. Our RNA-Seq and qRT-PCR results indicate that the expression levels of the key genes related to IAA signal transduction, including *AUX1*, were significantly upregulated in L28, and the expression levels of the *GH3* family protein genes were significantly upregulated in XLZ77. These findings indicate that auxin signal transduction is positively regulated in L28 but negatively regulated in XLZ77. Furthermore, IAA content is higher in L28 than in XLZ77. Together, these results show that auxin promotes the elongation of fruiting branches in cotton. Therefore, we predicted that IAA, when maintained at a certain level and experiencing upregulated signaling, may be a hormone trigger of internode elongation.

The phytohormone ET plays roles in various physiological processes throughout the life cycle of a plant [35]. Ethephon has highly significant effects on plant height [36], ear height [37, 38] and internode length [39]. Reduced plant height might be due to decreased internode length in response to the application of ethephon [40]. Moreover, elevated tissue ET concentrations inhibit longitudinal cell extension and thus stem growth [41, 42]. ET inhibits the elongation of maize internodes by inhibiting the longitudinal elongation of cells [43]. ET signal transduction supposedly follows a “linear” pathway, with membrane-bound receptors at the beginning, multiple positive and negative regulators in between, and TFs at the end of the chain [44]. *EIN2* is a positive regulator of the pathway, which plays a major role in the ET response [45]. Members of the *EIN3* family are involved in a regulatory cascade and stimulate the transcription of other TFs such as *ERF1* [46], which is a member of the *ERF* family of TFs [47]. These TFs have been shown to act as activators or repressors of additional downstream ET-responsive genes [48]. Our RNA-Seq and qRT-PCR results indicate that the genes related to *EIN2*, *EIN3* and *ERF1/2* were significantly highly expressed in XLZ77. This finding indicates that ET signal transduction is positively regulated in XLZ77. In addition, the content of ACC (the precursor of ET) in XLZ77 is higher than that in L28. Together, these data suggest that ET may act as a negative regulator of the internode elongation of fruiting branches in cotton.

The phytohormone JA plays essential roles in plant growth and development [49]. Methyl jasmonate (MeJA) inhibits root growth in some plant species. High concentrations of JA (25 μmol/L) can also inhibit plant growth [50]. JA inhibits the expression of cell cycle-related proteins and thus inhibits cell division [51]. JA inhibits the growth of the main roots of *Arabidopsis thaliana* by inhibiting cell division activity of the root meristem [52]. Our RNA-Seq and qRT-PCR results showed that positively regulated factors of JA signal transduction, including *JAR1*, *COI1* and *MYC2*, were significantly upregulated in XLZ77. This indicates that JA signal transduction is positively regulated in XLZ77 but negatively regulated in L28. Furthermore, the JA content in XLZ77 was higher than that in L28. Together, these results indicate that JA can inhibit the internode elongation of fruiting branches in cotton.

CKs affect many processes in plants, the most important of which are probably cell division and proliferation in the SAM, which are responsible for the production of all aboveground organs [53, 54]. Within the context of plant development, the CK phytohormone play key regulatory roles in the meristems (stem cell centers) and generally positively regulate the SAM by stimulating cell division [55]. CK oxidative decomposition catalyzed by *CKX* is an important mechanism for regulating the dynamic balance of CK content [56]. Our RNA-Seq and qRT-PCR results indicate that the expression of *CKX*-related genes (CK inactivating genes) was higher in XLZ77 than in L28. Because of the low level of CKs, the cell division process was suppressed in XLZ77. Furthermore, the content of ZT was higher in L28 than in XLZ77. Taken together, these data suggested that CK may act as a positive regulator in the internode elongation of fruiting branches in cotton.

**Fig. 6:**
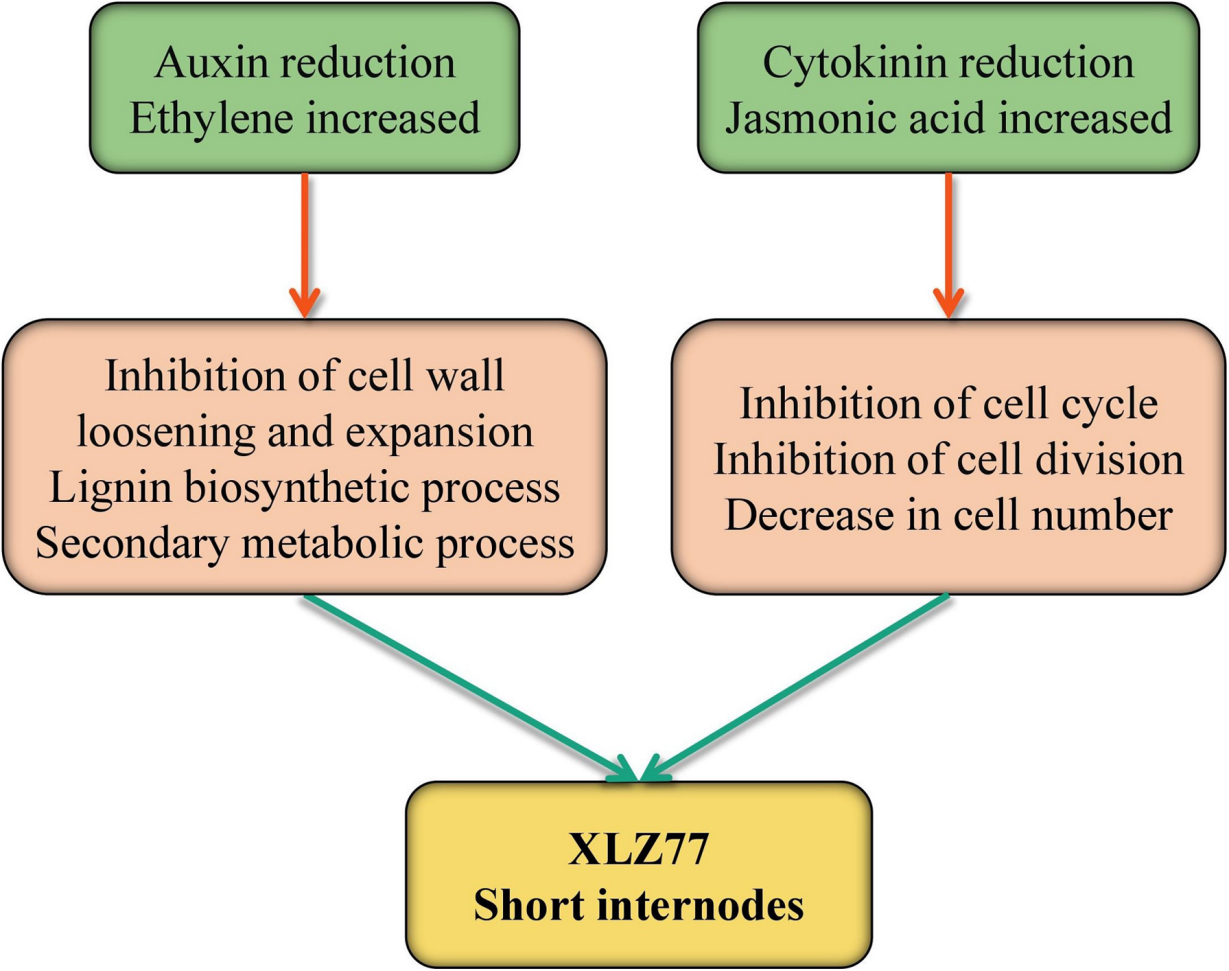
Suggested model for plant hormone-mediated regulation of internode elongation in cotton

In summary, we propose a hypothetical model to explain the role of phytohormones in the internode elongation of fruiting branches in cotton (Fig. 6). In XLZ77, upregulated ET and JA can inhibited the elongation and division of cells, resulting in fewer cells and a slow increase in size, thus leading to a relatively short internode length of the fruiting branches. However, the content and signal transduction of both auxin and CK were downregulated, which resulted in slowed cell elongation, expansion and cell division; thus, the number and volume of cells increased slowly, eventually inhibiting the internode elongation of the cotton fruiting branches.

### Regulatory role of TFs in the internode elongation of cotton fruiting branches

TFs are regulatory proteins that can activate or repress the transcription of multiple target genes in living organisms, and TF-mediated gene expression regulatory networks play important roles in plant growth and development. Plant growth and development can be regulated by regulating a series of TFs. In the present study, 2067 TFs were significantly enriched among all genes between the two genotypes. Among the various TFs, the TFs *WRKY*, *ERF* and *bHLH* constituted the top three largest families who members were active between these two genotypes. In addition, most of the TFs were significantly upregulated in XLZ77.

*WRKY* proteins compose a large superfamily of transcriptional regulators that are involved primarily in various plant physiological programs. Members of the *WRKY* TF family play an indispensable role in plant root, stem and leaf formation and development. *OsWRKY78* can regulate both stem elongation and seed development [57]. Similarly, *AtWRKY71* regulates branching development in *Arabidopsis thaliana* [58]. *GhWRKY15* not only contributes to the alteration of defense resistance to both viral and fungal infections but also affects plant growth and development, especially stem elongation [59]. Our results showed that most of the 224 *WRKY*-related genes were upregulated in XLZ77. These findings indicate that *WRKY* TFs may play negative regulatory roles in the internode elongation of cotton branches.

The typical biological effect of ET is the inhibition of both the elongation and growth of stems and roots and the promotion of stem and root lateral growth. The *ERF* family of TFs, which is present only in the plant kingdom, is characterized by the presence of a highly conserved DNA-binding domain [60] that can positively regulate the signal transduction pathway of ET. Moreover, some rice *ERFs* have been reported to plays roles in regulating internode elongation [61]. Another *ERF* gene, *SUB1A*, restricts plant elongation at the seedling stage during flash floods [62]. The rice *AP2/ERF* protein *OsEATB* restricts internode elongation by downregulating a GA biosynthesis gene [63]. Our data showed that 399 genes related to *ERF* TFs were enriched, and approximately 2/3 of these genes were highly expressed in XLZ77. Thus, *ERF* TFs may play a negative role in the internode elongation of fruiting branches in cotton.

*bHLH* TFs compose a large family within the plant genome. These TFs play important roles in plant growth and development, nutrient absorption, biosynthesis, signal transduction, etc. [64]. *ILI1* and *PRE1* interact with the *bHLH* protein *IBH1* (*ILI1* binding *bHLH*), whose overexpression causes erect leaves in rice and dwarfism in *Arabidopsis* [65]. The *LAX* (*OsHLH164*) gene is expressed at the boundary between the SAM and the region of new meristem formation; *LAX* genes have been identified as the main regulators of axillary meristem formation in rice [66]. Our results showed that 256 genes related to *bHLH* TFs were significantly enriched, and approximately 3/4 of these genes were highly expressed in XLZ77. These findings indicate that *bHLH* TFs may play a negative regulatory role in fruiting branch internode elongation in cotton.

In summary, we can assume that the *WRKY*, *bHLH* and *ERF* TFs mainly inhibit the internode elongation of cotton fruiting branches. The upregulation of these TFs in XLZ77 may be the primary reason for the relatively short internodes in that variety. The differences in endogenous hormone content may be caused by mutations in TFs. In the future, we will attempt to locate these mutation sites by map-based cloning to further explore the mechanism of internode elongation.

## Conclusions

In this study, we selected the fruiting branch internodes at three different stages from two cotton cultivars whose fruiting branch internode lengths significantly differed. A total of 76,331 genes were detected by transcriptome sequencing. The potential roles of a series of DEGs involved in the internode elongation of cotton were identified and analyzed by GO category and KEGG pathway analyses, qRT-PCR verification and hormone content determination. We found that auxin and CK are the positive regulators of internode elongation in cotton branches. In contrast, ET and JA may act as negative regulators of internode elongation in cotton branches. Furthermore, the *WRKY*, *ERF* and *bHLH* TFs were identified as important inhibitors of the elongation of internodes in cotton. In XLZ77 (a short-internode variety), the mass synthesis of ET and the amino acid conjugation of auxin led to the inhibition of plant cell elongation, while the increase in JA content and the degradation of CKs led to a slow rate of cell division, which eventually resulted in a phenotype that included relatively short internodes of fruiting branches. The results of this study not only provide gene resources for the genetic improvement of cotton plant architecture but also lay a foundation for improved understanding of the molecular mechanism of the internode elongation of cotton branches.

## Methods

### Plant material and sampling

In our study, two cotton genotypes—a short-internode cultivar (Xinluzhong77, XLZ77) and a long-internode cultivar (Lumianyan28, L28) —were used. The two genotypes were planted at the experimental station of the Institute of Cotton Research of the CAAS in Anyang City (Henan Province, China) on April 20, 2017, after which the plants were subjected to routine field management. The first internodes of the first, second and third branches from the top of the plants were collected at the cotton bud stage (July 6, 2017). A schematic diagram of the cotton sampling positions is shown in Additional file 12. Three biological replicates were included; the samples of each biological replicate were pooled from 10 plants, which were randomly selected to avoid any potential effects of position in the field. The three biological replicates were mixed together and then divided into two groups; one group was used for RNA isolation via Illumina sequencing and qRT-PCR analysis, and the other was used for endogenous hormone measurements. The samples were frozen immediately in liquid nitrogen and then stored at − 80 °C until use.

### RNA isolation, library construction and RNA-Seq

Total RNA was extracted using a Plant RNA Kit (Omega Bio-Tek, USA) according to the manufacturer’s protocol. RNA integrity and quantity were verified by RNase-free agarose gel electrophoresis and with a 2100 Bioanalyzer (Agilent Technologies, USA). Subsequent experiments were carried out with qualified RNA samples.

A sequencing library was constructed with a NEBNext1 Ultra™ Directional RNA Library Prep Kit for Illumina (NEB, USA) according to the manufacturer’s recommendations. Briefly, the total mRNA was isolated with oligo (dT) beads. All of the mRNA was cut into short fragments (200 nt) by adding a fragmentation buffer. First-strand cDNA was generated using random hexamer-primed reverse transcription. Second-strand cDNA was then synthesized by DNA polymerase I and RNase H. Afterward, the synthesized cDNA fragments were purified and then subjected to end pairing, the addition of a single “A” base, and ligation with Illumina adapters. The ligation products were subsequently size-fractioned by agarose gel electrophoresis, after which the fragments were excised for PCR amplification. The amplified fragments were sequenced using an Illumina HiSeq™ 4000 by Gene Denovo Co. (Guangzhou, China).

### Sequencing analysis and differential expression analysis

After sequencing, the reads with adapter sequences were removed. Reads with more than 10% N bases and low-quality (Q ≤ 20) reads with more than 50% bases were then removed from each data set to gain more reliable results. The alignment software TopHat2 (v2.1.1) was used to map the reads to the *Gossypium hirsutum* L*.* genome [67]. The number of mapped clean reads for each gene was then counted and normalized into reads per kilobase per million reads (RPKM) for calculating gene expression. When comparing two groups, edgeR was used to analyze DEGs to correct for multiple testing, and the FDR was calculated to adjust the threshold of the *p*-value. Genes with a minimum 2-fold difference in expression, |log2FC| ≥ 1 and FDR ≤ 0.05 were considered DEGs.

GO classification was performed via WEGO (http://wego.genomics.org.cn/cgi-bin/wego/index.pl), and the GO categorization results were expressed as 3 independent hierarchies for molecular function, biological process and cellular component. For each KEGG pathway, the numbers of DEGs were compared to the entire reference gene set by hypergeometric tests to determine the pathways enriched for differentially regulated genes. The *p*-values of the GO and KEGG enrichment analyses were adjusted using the Bonferroni correction, and a corrected p-value ≤0.05 was chosen as the threshold value for determining significantly enriched GO terms. With respect to KEGG enrichment analysis, pathways with an FDR value≤0.05 were considered enriched.

### Real-time PCR validation

Twelve genes that exhibited different expression patterns as revealed by RNA-Seq were selected for validation by qRT-PCR. Total RNA was extracted from the same samples that were used for sequencing. First-strand cDNA was synthesized using a Primer Script RT Reagent Kit (Takara Bio Inc., Shiga, Japan). The primer sequences used were designed with Primer Premier 5.0 software (Premier Biosoft International, Palo Alto, CA, USA) and synthesized by Sangon Biotech (Shanghai) Co., Ltd. The specific primers for the selected genes and the internal control gene (*UBQ*) are listed in Additional file 13. qRT-PCR was performed on a 7500 Fast Real-Time PCR System (Applied Biosystems, StepOnePlus, USA) using BCS® Wiz Universal SYBR Green qPCR Master Mix (Transgen Biotech, Beijing, China). The cotton *UBQ* gene was used as an internal standard to calculate relative fold differences based on comparative cycle threshold (2^-ΔΔCt^) values [68]. Then, ddH_2_O was then used as a nontemplate control. The qRT-PCR procedure was as follows: 2 μL of a 1/8 dilution of cDNA in H_2_O was added to 10 μL of SYBR Green PCR Master Mix; 0.4 μL of each primer and 7.2 μL of H_2_O were then added, and the final volume was brought to 20 μL. The qPCR program was as follows: 50 °C for 2 min; 95 °C for 10 min; followed by 40 cycles of 95 °C for 30 s, 56 °C for 30 s, and 72 °C for 30 s in 96-well optical reaction plates. Each real-time PCR was performed three times.

### Measurements of various hormones

To analyze the endogenous hormone contents, independent samples from XLZ77 and L28 were harvested, immediately frozen in liquid nitrogen and then stored at − 80 °C for further determination. Each sample was prepared in triplicate. Endogenous IAA, ZT, JA and ACC contents were determined using an ultra-performance liquid chromatography-electrospray ionization-tandem mass spectrometry (UPLC-ESI-MS/MS) system [69].

### Statistical analysis

Significant differences between values were determined using one-way ANOVA with the Tukey test at a significance level of α = 0.01 in Excel software. All expression analyses were performed for three biological replicates. All reported values represent the arithmetic averages of three replicates, and the data are expressed as the mean plus or minus standard deviation (means ± SD).

## Additional files

Additional file 1: Detailed information on the obtained reads via RNA-Seq. (XLS 20 kb)

Additional file 2: Expression of all genes in the 18 samples. (XLS 64722 kb)

Additional file 3: Detailed information on the number of DEGs between each pair of compared groups. (JPG 275 kb)

Additional file 4: List of KEGG pathways enriched in each comparison. (XLS 163 kb)

Additional file 5: List of genes associated with various aspects of hormone homeostasis. (XLS 1631 kb)

Additional file 6: List of genes related to hormone signal transduction in Fig. 2. (XLS 305 kb)

Additional file 7: Additional figure showing the RT-PCR validation of three genes related to plant hormone signal transduction. (JPG 173 kb)

Additional file 8: List of enriched GO categories in each comparison. (XLS 12995 kb)

Additional file 9: List of the DEGs expressed in 10 modules. (XLS 9181 kb)

Additional file 10: List of enriched GO categories in 10 modules. (XLS 2393 kb)

Additional file 11: List of the expressed TF genes. (XLS 4511 kb)

Additional file 12: Schematic diagram of the sampling position of cotton. (JPG 38 kb)

Additional file 13: List of primers used for qRT-PCR. (DOC 44 kb)

## Data Availability

The datasets supporting the conclusions of this article are included within the article and its additional files.

## Abbreviations

ABA: abscisic acid
*AUX1*: auxin influx carrier
*bHLH*: basic helix-loop-helix
*CAD*: cinnamyl alcohol dehydrogenase
*CCOAOMT1*: S-adenosyl-L-methionine-dependent methyltransferase superfamily protein
CK: cytokinin
*CKX*: cytokinin oxidase/dehydrogenase
*COI1*: an insensitive mutant of coronatine, jasmonate SCF-COI1 receptor complex
*CTL2*: chitinase-like protein 2
*CTR1*: serine/threonine protein kinase
DEG: differentially expressed gene
*EIN2/3*: ethylene-insensitive protein
*ERF*: ethylene response factor
ET: ethylene
*ETR*: ethylene receptor
*EXP*: expansion
*F5H*: ferulic acid 5-hydroxylase
FDR: false discovery rate
GA: gibberellin
*GH3*: gretchenhagen-3
GO: Gene Ontology
IAA: indole-3-acetic acid
JA: jasmonic acid
*JAR1*: jasmonate synthetase
KEGG: Kyoto Encyclopedia of Genes and Genomes
*LAC*: laccase/diphenol oxidase family protein
LOF: loss of function
*MAPKKK*: mitogen-activated protein kinase kinase kinase
NCBI: National Center of Biotechnology Information
RNA-Seq: RNA Sequencing
RPKM: reads per kilobase per million reads
SAM: shoot apical meristem
*SBH2*: sphingoid base hydroxylase 2
TF: transcription factor
*WAT1*: the walls are thin1
*XTH*: xyloglucan endotransglucosylase /hydrolase
*ZIM*: ZIM domain protein
ZT: zeatin
