# Supplementary figures and images for: Transcriptome analysis and identification of genes associated with fruiting branch internode elongation in upland cotton
Source: BMC Plant Biol. 2019 Oct 7;19:415. doi: 10.1186/s12870-019-2011-8 (PMC6781417; doi:10.1186/s12870-019-2011-8)

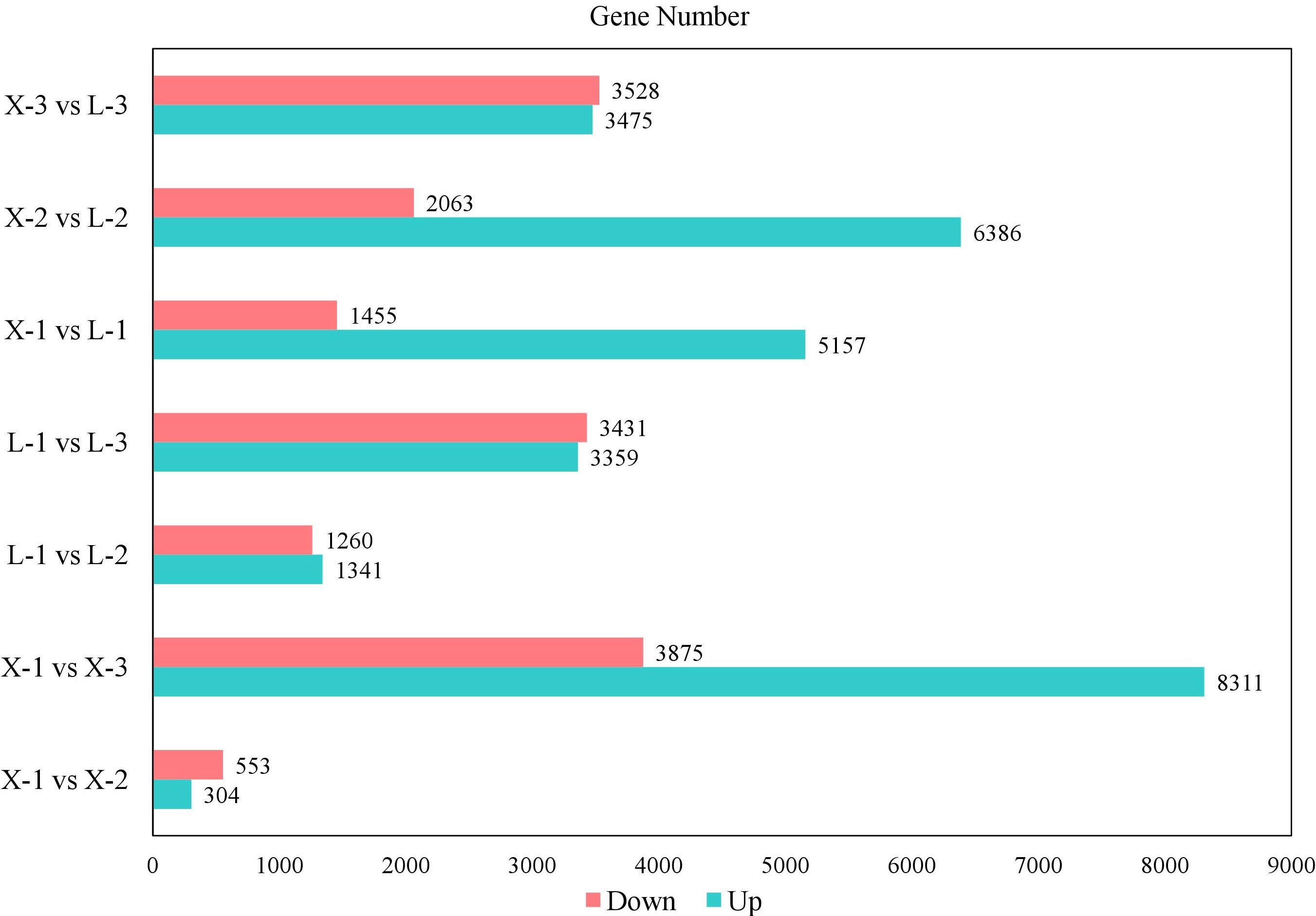

Supplement: Supplementary file 3 — Detailed information on the number of DEGs between each pair of compared groups. (JPG 275 kb) [file 12870_2019_2011_MOESM3_ESM.jpg]

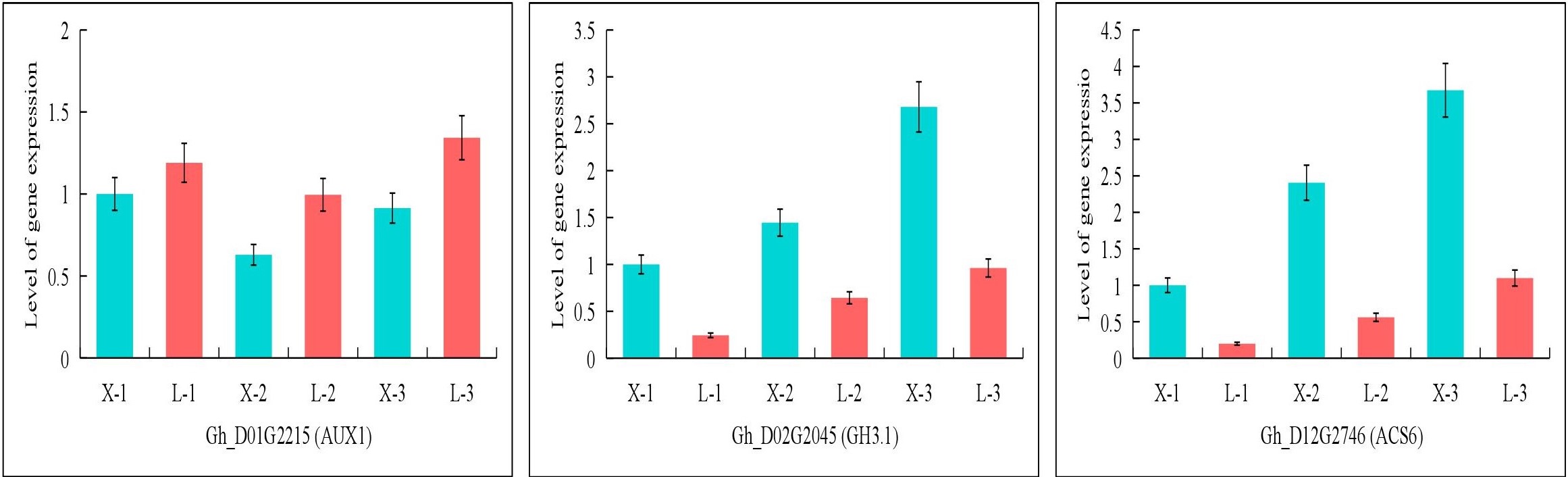

Supplement: Supplementary file 7 — Additional figure showing the RT-PCR validation of three genes related to plant hormone signal transduction. (JPG 173 kb) [file 12870_2019_2011_MOESM7_ESM.jpg]

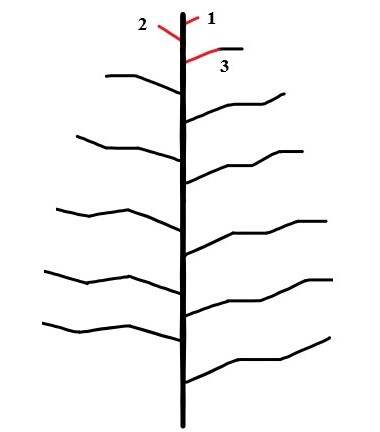

Supplement: Supplementary file 12 — Schematic diagram of the sampling position of cotton. (JPG 38 kb) [file 12870_2019_2011_MOESM12_ESM.jpg]
