# Supplementary material for: Transcriptome analysis and identification of genes associated with fruiting branch internode elongation in upland cotton
Source: BMC Plant Biol. 2019 Oct 7;19:415. doi: 10.1186/s12870-019-2011-8 (PMC6781417; doi:10.1186/s12870-019-2011-8)
Supplement: Supplementary file 13 — List of primers used for qRT-PCR. (DOC 44 kb) [file 12870_2019_2011_MOESM13_ESM.doc]

Table The specific primers for the selected genes and internal control gene (UBQ)

| Gene | Primer | Sequence | Gene ID |
| --- | --- | --- | --- |
| Gh_D01G2215 (AUX1) | F | TGCCGTCACCGTGGAAATTA | Gh_D01G2215 |
|  | R | GAAGTACAGCGGTGTGCAAG |  |
| Gh_D02G2045 (GH3.1) | F | GGAAATCGGGAAAGAATAC | Gh_D02G2045 |
|  | R | CTCCTCACGAAATGGAACTG |  |
| Gh_A03G1628 (GH3.1) | F | AGGAAACTGATGCCAACTATT | Gh_A03G1628 |
|  | R | TGAAATGCTCGCTCTTGTAG |  |
| Gh_A11G2892 (EIN2) | F | AGTTAACGAGGCAGCTGATG | Gh_A11G2892 |
|  | R | GGCAGCTAATTGACGCCTTG |  |
| Gh_A13G2005 (EIN3) | F | CAGGTCGAAATGGATGCACTA | Gh_A13G2005 |
|  | R | TTGCTTGTTCTTGCGACTGG |  |
| Gh_D11G1357 (ERF1B) | F | GTTGCTGGGACGACCTTCTT | Gh_D11G1357 |
|  | R | CGAGTCTCTAATCTCGGCGG |  |
| Gh_D04G0642 (COI1) | F | TGGCTTCATGAGCTTGCAG | Gh_D04G0642 |
|  | R | CCAAACGGCATAACCTCGGA |  |
| Gh_A08G1412 (MYC2) | F | TTCTATTCCTGCTGGAAATCATCAC | Gh_A08G1412 |
|  | R | TTCCCATTCCTCACACTGCT |  |
| Gh_D08G1403 (JAR1) | F | CAAAATCTGGGCCTCAATGGC | Gh_D08G1403 |
|  | R | GTGAGAATCGGAGAGGACGAA |  |
| Gh_A12G2673 (ACS6) | F | GCTGTCCGATGATGATTTTGT | Gh_A12G2673 |
|  | R | GAACCGATACCGACTTGAGAA |  |
| Gh_D12G2746 (ACS6) | F | AAGTCGGTATCGGTTCGTTGA | Gh_D12G2746 |
|  | R | AATCACTCGCCACAAGTCCAT |  |
| Gh_D06G0885 (CKX7) | F | TGCAACAACGACGATCCAGT | Gh_D06G0885 |
|  | R | AACAGCCAGTTCAAGGCAGT |  |
| UBQ | F | AGCTCGGATACGATTGATAACG |  |
|  | R | GAAGACGAAGAACAAGGGGAAG |  |
